# Morphological and Functional Changes of Roof Plate Cells in Spinal Cord Development

**DOI:** 10.3390/jdb9030030

**Published:** 2021-07-30

**Authors:** Takuma Shinozuka, Shinji Takada

**Affiliations:** 1 Exploratory Research Center on Life and Living Systems, National Institutes of Natural Sciences, 5-1 Higashiyama, Myodaiji, Aichi, Okazaki 444-8787, Japan; 2 National Institute for Basic Biology, National Institutes of Natural Sciences, 5-1 Higashiyama, Myodaiji, Aichi, Okazaki 444-8787, Japan; 3 Department of Basic Biology, School of Life Science, The Graduate University for Advanced Studies (SOKENDAI), 5-1 Higashiyama, Myodaiji, Aichi, Okazaki 444-8787, Japan

**Keywords:** Wnt, roof plate, spinal cord, morphogenesis, central canal, dorsal collapse, dorsal median septum, neural crest

## Abstract

The most dorsal region, or roof plate, is the dorsal organizing center of developing spinal cord. This region is also involved in development of neural crest cells, which are the source of migratory neural crest cells. During early development of the spinal cord, roof plate cells secrete signaling molecules, such as Wnt and BMP family proteins, which regulate development of neural crest cells and dorsal spinal cord. After the dorso-ventral pattern is established, spinal cord dynamically changes its morphology. With this morphological transformation, the lumen of the spinal cord gradually shrinks to form the central canal, a cavity filled with cerebrospinal fluid that is connected to the ventricular system of the brain. The dorsal half of the spinal cord is separated by a glial structure called the dorsal (or posterior) median septum. However, underlying mechanisms of such morphological transformation are just beginning to be understood. Recent studies reveal that roof plate cells dramatically stretch along the dorso-ventral axis, accompanied by reduction of the spinal cord lumen. During this stretching process, the tips of roof plate cells maintain contact with cells surrounding the shrinking lumen, eventually exposed to the inner surface of the central canal. Interestingly, Wnt expression remains in stretched roof plate cells and activates Wnt/β-catenin signaling in ependymal cells surrounding the central canal. Wnt/β-catenin signaling in ependymal cells promotes proliferation of neural progenitor and stem cells in embryonic and adult spinal cord. In this review, we focus on the role of the roof plate, especially that of Wnt ligands secreted by roof plate cells, in morphological changes occurring in the spinal cord.

## 1. Roof Plate Functions in the Early Developmental Stage of Spinal Cord

### 1.1. Generation of Trunk Neural Crest Cells

**Figure 1:**
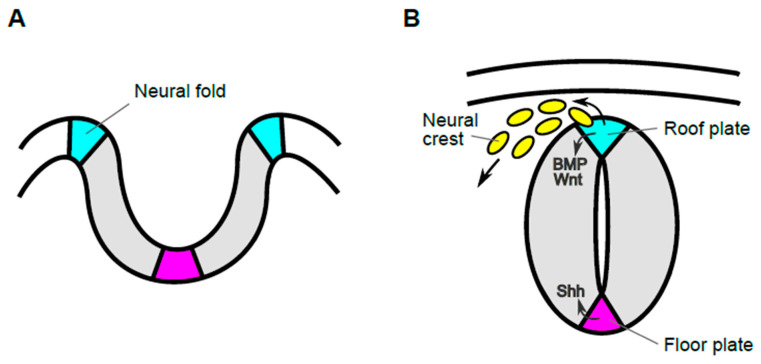
Transition in morphology of neural tissue and functions of cells in the most dorsal region during early developmental stages. Schematic images of transverse sections of developing spinal cord before (**A**) and after (**B**) closure of the neural tube during mouse development. The edge of the neural plate elevates and becomes the neural fold (cyan), from which neural crest cells are generated (**A**). Then, the lateral tips of the neural fold fuse, generating the neural tube (**B**). Cells in the most dorsal region of the neural tube, or roof plate (cyan), act as progenitors of migrating neural crest cells (yellow) and also as the organizing center for dorsal neuroepithelial cells. Roof plate cells secrete signaling molecules such as Wnt and BMP, which govern development of both neural crest and dorsal neuroepithelial cells. By contrast, cells in the most ventral region, the floor plate (magenta), act as the ventral organizer, by producing Shh.

In the beginning of neural development, the neural plate gradually invaginates and its lateral edges are transformed into the neural fold. The appearance of the neural fold is probably the first morphological indication of the dorsal region of neural tissues. Then, the tips of the neural fold fuse, resulting in formation of the neural tube, which develops into the brain in the head and the spinal cord in the trunk. In the mouse, anterior neural tube is generated by this process, called primary neurulation (Figure 1). On the other hand, the posterior neural tube is formed by a process called secondary neurogenesis, in which the neural tube is formed from precursors in the tail bud, followed by condensation of the mesenchyme and subsequent epithelialization [1]. After these processes, neuroepithelial cells adjacent to the lumen proliferate rapidly and differentiate into several distinct types of neuronal and glial cells. Roof plate cells are located in the most dorsal part of developing spinal cord and serve as the organizing center for surrounding neuroepithelial cells, promoting their proliferation and specification [2]. Prior to functioning as an organizing center, these cells give rise to neural crest cells, which migrate to many different tissues, where they give rise to neurons and glial cells of the sensory, sympathetic, and parasympathetic nervous systems, pigment-containing cells of the epidermis, and chromaffin cells of the adrenal gland [3,4,5,6]. Thus, cells in the roof plate, have markedly different roles and their behaviors are dynamic in early embryonic stages.

During development of the neural tube, premigratory neural crest cells first exist in the neural fold and undergo delamination and epithelial-mesenchymal transition to become migratory neural crest cells [7]. However, the timing and mechanism of fate determination in these neural crest cells remains controversial. Lineage tracing analysis using *R26R-Confetti* mice revealed that the vast majority of individual premigratory and even migratory neural crest cells are multipotent. Furthermore, in some clones with labeled progeny cells among neural crest derivatives, labeled progeny cells are retained in the dorsal neural tube, suggesting an asymmetric cell division of premigratory neural crest cells in the dorsal neural tube [6]. By contrast, lineage tracing analysis using avian embryos after electroporation with GFP reporter showed that pre-migratory neural crest cells generate progeny in single, rather than multiple derivatives, in most cases where delaminated neural crest cells are labeled. In these cases, no labeled cells remained in the neural tube [8]. This result with avian embryos suggests that premigratory neural crest cells are a distinct population from cells that remain in the neural tube, such as roof plate cells, which act as the organizing center. In addition, this transition from neural crest to roof plate accompanied by loss of responsiveness to BMP signaling in dorsal spinal cord [8,9,10]. These discrepancies may be due to differences in labeling techniques, in the stage and location of labeling, or in mechanisms of lineage segregation between mammalian and avian systems.

During development of neural crest cells, cells in the most dorsal region of the spinal cord produce secreted ligands such as BMP and Wnt [2,11,12] (Figure 1B). Several lines of evidence indicate that Wnt ligands, which activate Wnt/β-catenin signaling, are required for generation of neural crest cells. In the spinal cord of mouse embryos, two Wnt ligands, *Wnt1* and *Wnt3a*, are specifically expressed in roof plate cells [13,14,15]. These Wnt ligands activate a common signaling pathway, the Wnt/β-catenin pathway, and are functionally redundant in the dorsal spinal cord. Thus, neither *Wnt1* nor *Wnt3a* single KO mutants show any obvious defects in dorsal spinal cord development, although *Wnt1* KO mutant impairs the development of midbrain and cerebellum and *Wnt3a* KO mutant exhibits severe truncation of axis elongation [14,16,17,18]. On the other hand, *Wnt1* and *Wnt3a* double-mutant embryos exhibit a reduction of neural crest cell number and a marked deficiency of neural crest derivatives [19]. Similarly, Wnt/β-catenin signaling can induce and is required for neural crest formation in other species [20,21,22,23,24]. 

In addition to formation of neural crest cells, Wnt/β-catenin signaling also promotes segregation of sub-lineages of neural crest cells [25,26,27,28,29]. For instance, conditional loss of function of β-catenin in the mouse roof plate reduces melanocytes and *Ngn2*-positive sensory lineage cells, resulting in impaired formation of neurons and glial cells in dorsal root ganglia (DRG) [26]. On the other hand, studies in which β-catenin is activated at different time points in neural crest differentiation suggest the importance of this signaling in determining the fate of the neural crest sub-lineage [27,29]. In zebrafish, studies of gain- and loss-of-function of Wnt/β-catenin signaling in pre-migratory cranial neural crest cells also indicate the importance of this signaling in determining the fate of the neural crest sub-lineage [25]. Furthermore, in addition to Wnt/β-catenin signaling, ligands of the BMP family are also involved in fate decision of neural crest cells. For instance, late emigrating neural crest cells in the roof plate are restricted to a sensory fate by *Gdf7* [30], and BMP2 antagonizes sensory specification induced by Wnt signaling [31]. 

### 1.2. Specification of Dorsal Interneurons

Direct evidence showing the requirement for roof plate cells in specification of dorsal neuroepithelial cells comes from genetic ablation of roof plate cells with *Gdf7-DTA.* Progenitors of dorsal interneurons are subclassified as dI1 to dI6, in dorsal-to-ventral order in developing spinal cord [32]. This ablation causes loss of progenitors of dorsal interneurons dI1-3 and compensatory occupation of a dorsal position by dI4-6 [33]. This specification, as well as proliferation of dorsal neuroepithelial cells, is regulated by roof plate-derived Wnt and BMP family proteins. For instance, *Wnt1* and *Wnt3a* double-mutant embryos exhibit impaired proliferation and specification of cells in the dorsal spinal cord [34]. A similar phenotype is also observed in mutant embryos in which components of the Wnt/β-catenin pathway, including *Wntless* and *β-catenin*, are impaired [35,36]. In addition, activation of Wnt/β-catenin signaling in developing spinal cord indicates that this signaling can regulate specification and proliferation of neuroepithelial cells [36,37,38]. In terms of Wnt-mediated proliferation, it has been proposed that several Wnt ligands expressed in the dorsal spinal cord generate a proliferation gradient along the dorso-ventral axis [39]. 

BMP family proteins, including BMP4, BMP6, BMP7, and Gdf7, are also expressed in the surface ectoderm and the dorsal spinal cord and are involved in differentiation of dorsal interneurons [40,41,42,43,44]. Manipulation of BMP signaling can promote differentiation of dorsal interneurons in vitro [45,46]. Genetic analyses with mutant embryos showed that *Gdf7* is required in formation of dI1 interneurons [42] and that *Bmp7* is similarly essential for several subtypes of dorsal interneurons [47]. In contrast, inhibitory Smad6 and Smad7 are expressed in the neural tube, restricting the action of BMP signaling in the dorsal neural tube [48]. *Bmp7* and *Gdf7* are also required for dI1 axon growth [49]. Since activation of Wnt/β-catenin signaling in roof plate induces expansion of BMP signaling activity in dorsal spinal cord [50], combinatorial Wnt and BMP signaling appears to regulate dorsal interneuron specification and proliferation. Moreover, recent study revealed that Notch signaling in dorsal neural tube is also required for roof plate and dI1 formation. Loss of Notch function causes missing of roof plate and dI1 with a compensatory expansion of dI2 [51]. Taken together, there are several lines of evidence to show that signaling molecules secreted from roof plate cells are critical in neural crest formation and interneuron specification. On the other hand, there are few studies examining the role of roof plate-derived ligands in later developmental stages, as discussed below. 

## 2. Morphological Transformation of the Spinal Cord and Central Canal Formation in Mouse Development

### 2.1. Morphological Transformation of the Spinal Cord

**Figure 2:**
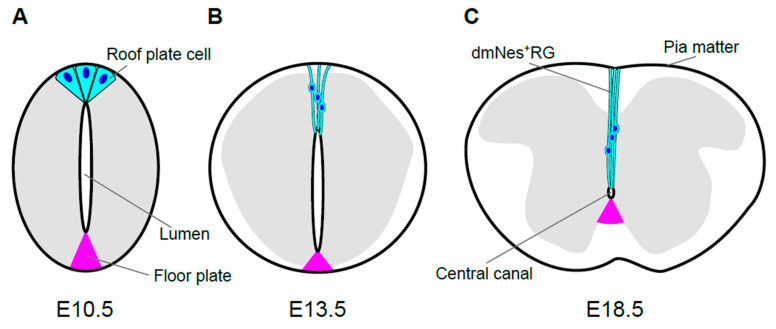
Morphological transformation of the mouse spinal cord. Schematic images of spinal cord morphology and roof plate shape are shown at E10.5 (**A**), E13.5 (**B**), and E18.5 (**C**) during mouse development. The gray matter of the spinal cord is shown in gray. Roof plate cells, or their descendants, and their nuclei are shown in cyan and blue, respectively. The size and morphology of spinal cord change dramatically during development. In accordance with these changes, morphology of roof plate cells also transforms. In mouse embryos, the apical sides of roof plate cells gradually constrict, such that roof plate cells assume a wedge-shaped form at E10.5 (**A**). Then, the lumen of the spinal cord shrinks gradually. The reduction of lumen size is caused by gradual attrition of neuroepithelial cells adjacent to roof plate cells. As a result, cell reduction from the surface of the lumen proceeds progressively in a dorsal-to-ventral manner known as dorsal collapse. Accompanying the dorsal collapse and reduction of the lumen, roof plate cells are stretched along the dorso-ventral axis and line up along the midline, resulting in morphological transformation. Compared to the dorsal side, reduction of the ventral ventricular zone and transformation of floor plate (magenta) are comparatively smaller (**B**). The stretched roof plate cells are also known as dorsomedial Nestin-positive radial glia (dmNes^+^RGs). At E18.5, the diameter of the lumen finally shrinks to roughly a few cell diameters, resulting in the central canal, a cavity filled with cerebrospinal fluid and connected to the ventricular system of the brain. Quiescent neural stem cells locate around the central canal. At this stage, roof plate cells (dmNes^+^RGs) are stretched, maintaining contact with the inner surface of the central canal and also with the pia mater, which covers the outer surface of the spinal cord (**C**).

During development of spinal cord, its size and morphology change dramatically. Neuroepithelial cells proliferate and give rise to migrating cells that accumulate around the original layer of neuroepithelial, or neuroprogenitor, cells. This accumulation results in formation of the mantle zone and the layer of neuroprogenitor cells remaining along the lumen, which is now called the ventricular layer. Cells in the mantle zone differentiate into neurons and glia, and these neurons are interconnected and extend their axons to the lateral region of the spinal cord, known as the marginal zone [11]. The mantle zone, which will form the gray matter of the spinal cord, gradually becomes a butterfly-shaped structure, surrounded by the marginal zone, which will form the white matter (Figure 2C). The spinal cord, as well as the brain, is surrounded by three layers of meninges, the pia mater, the arachnoid mater, and the dura mater. As the spinal cord develops, all three layers are generated from a mesenchymal sheath on the surface of the developing spinal cord, called the primary meninx [52]. 

### 2.2. Morphological Transformation of the Lumen

Contemporaneously with morphological transformation of the spinal cord, the lumen of the spinal cord, which is surrounded by the ventricular layer, gradually diminishes in size and finally becomes the central canal, a cavity filled with cerebrospinal fluid (CSF) that is connected to the ventricular system of the brain. In mouse embryos, this reduction starts on approximately embryonic day 13.5 (E13.5; Figure 2B). Prior to its reduction, the lumen extends over almost the entire dorsoventral axis of the spinal cord. The size of lumen, i.e., the dorso-ventral length of the ventricular layer, is dramatically reduced between E13.5 and E15.5. By E17.5 and E18.5, the diameter of the lumen has shrunk to roughly a few cell diameters, resulting in the central canal [53,54] (Figure 2).

The central canal is lined with the ependymal layer, which is composed of several distinct cell types, including ciliated ependymal cells, tanycytes (a subpopulation of radial glia), and CSF-contacting, neuron-like cells [55,56,57,58]. Although the functions of these cell types remain to be determined, some of them are likely responsible for homeostatic regulation of CSF in adults [59,60]. Moreover, the ependymal layer includes quiescent stem cells, which generate progeny that undergo glial fates after injury to the spinal cord [55,61,62]. 

Of note, at the dorsal pole of the central canal, neuron-like cells with extensive projections are observed. These cells express Nestin, and their projections extend from the apical side facing the central canal toward the superficial regions of the spinal cord, as far as the meninges [63,64,65]. These cells are referred to as dorsal midline Nestin ^(+)^ radial glia (dmNes^+^RG) [65]. 

### 2.3. Origin of Ependymal Layer

Lineage-tracing analyses reveal that most cells comprising the ependymal layer are from the ventral ventricular zone, especially in the subdomains called p2 and pMN [66,67,68,69]. Conditional knock-out of components of Shh signaling, including *Shh* and *Smo,* reveals that Shh signaling is required for formation of the ependymal zone [69]. By contrast, dmNes^+^RG cells which are derived from the roof plate and cells at the ventral pole of the ependymal layer are from the floor plate [54,70]. Thus, cells forming the ependymal layer are heterogenous in origin and reduction of the lumen does not progress proportionally along the dorso-ventral axis.

### 2.4. Regulatory Mechanisms Governing Lumen Reduction

Reduction of lumen size is caused by gradual attrition of the neuroepithelial cell population in the ventricular zone (Figure 2). Importantly, this process is promoted by a morphological phenomenon known as dorsal collapse [54,65]. In dorsal collapse, cell reduction proceeds progressively in a dorsal-to-ventral manner in the ventricular zone. In this process, cells adjacent to the dorsal midline down-regulate apical polarity proteins and delaminate in a stepwise manner. The loss of polarity and delamination can be promoted by a secreted form of Crumbs2 produced by dmNes^+^RGs [65]. In addition, loss of apical polarity protein, such as pard6γb, is involved in this process, because loss of *pard6γb* disrupts dorsal collapse and lumen reduction in zebrafish [71,72]. Moreover, ventricular layer attrition is accompanied by reduction of cell proliferation in the ventricular layer but not apoptosis [54]. Compared to the dorsal side, reduction of the ventral ventricular zone is comparatively smaller, but apparent over time. This ventral reduction may be pronounced by migration of glial cells differentiated from neuroepithelial cells in the ventricular layer [54]. 

## 3. Development of Roof Plate Cells in Formation of the Central Canal

### 3.1. Formation of dmNes^+^RGs

As mentioned above, roof plate and floor plate cells remain at the dorsal and ventral poles of the ependymal layer, respectively. Labeling of zebrafish roof plate cells, as well as tracing of roof plate cells using mouse embryos carrying *Wnt1-creERT*, revealed that roof plate cells are actually elongated along the dorso-ventral axis and transformed into dmNes^+^RGs, accompanying the reduction of the lumen [35,72]. Low-frequency labeling of roof plate cells, which enables the morphology of each cell to be distinguished, revealed that dmNes^+^RGs contact the surface of the ependymal layer. dmNes^+^RGs also maintain contact with the outer surface of the spinal cord, the pia mater. Thus, it seems probable that roof plate cells are stretched, maintaining contact with the central canal and the pia mater (Figure 2C). dmNes^+^RGs eventually become part of the dorsal (or posterior) median septum, a thin, dense septum dividing the dorsal side of the spinal cord [35]. In contrast, floor plate cells do not exhibit dynamic morphological changes (Figure 2). Rather, only a subset of floor plate cells is retained around the central canal, whereas other floor plate cells separate from the ependymal layer during reduction of the lumen [54].

### 3.2. Morphology and Roles of dmNes^+^RGs

Along the stretching of dmNes^+^RGs, cytoskeletal structures are well developed. Electron microscopic analysis revealed enrichment of intermediate filament structure during this process [53,73]. This is consistent with enrichment of Nestin, which is a component of intermediate filaments, and directional organization of actin filaments in this process [35,63]. In zebrafish, inhibition of Zic6 or Rock impairs the stretching morphogenesis of roof plate with disruption of the actin cytoskeleton [72,74]. Thus, these cytoskeletal structures apparently contribute to formation of the physically robust structure of the dorsal median septum.

In the dorsal median septum, dmNes^+^RGs seem to act as a physical and molecular barrier, preventing decussation of developing long tracts of commissural axons [75,76]. In addition, dmNes^+^RGs apparently serve several different functions. For instance, *dreher* (*Lmx1a*-deficient) mice, which impair roof plate formation, show that dmNes^+^RGs regulate growth of long-range dorsal column axons [77,78]. Furthermore, as described below, dmNes^+^RGs promote proliferation of ependymal cells by producing Wnt ligands [35,70]. Thus, in addition to serving as a signaling center in early spinal cord development, roof plate has additional roles in later spinal cord development [10].

## 4. Wnt Signaling in Morphological Transformation of Roof Plate Cells

### 4.1. Expression of Wnt Ligands and Activation of Wnt Signaling

As described above, *Wnt1* and *Wnt3a*, which are secreted by roof plate cells, participate in development of neural crest cells and dorsal interneurons [19,34]. Whereas expression of *Wnt1* and *Wnt3a* mRNA is detected in the roof plate of developing mouse spinal cord until E12.5 [15,79], using in situ hybridization with a digoxygenin-based probe, it had been difficult to judge whether expression of these Wnt genes is maintained after E12.5, because roof plate cells become long and thin. However, recent immunostaining analysis revealed that expression of both Wnt1 and Wnt3a proteins remains in elongating roof plate cells, dmNes^+^RGs, after E13.5 [35]. This persistent expression was also confirmed with knock-in mouse embryos in which endogenous *Wnt3a* is replaced by *egfp-Wnt3a.* Consistent with Wnt expression in dmNes^+^RGs, activation of Wnt/β-catenin signaling is evident in dmNes^+^RGs [35,70]. 

### 4.2. Functions of Wnt Ligands Secreted by dmNes^+^RGs

**Figure 3:**
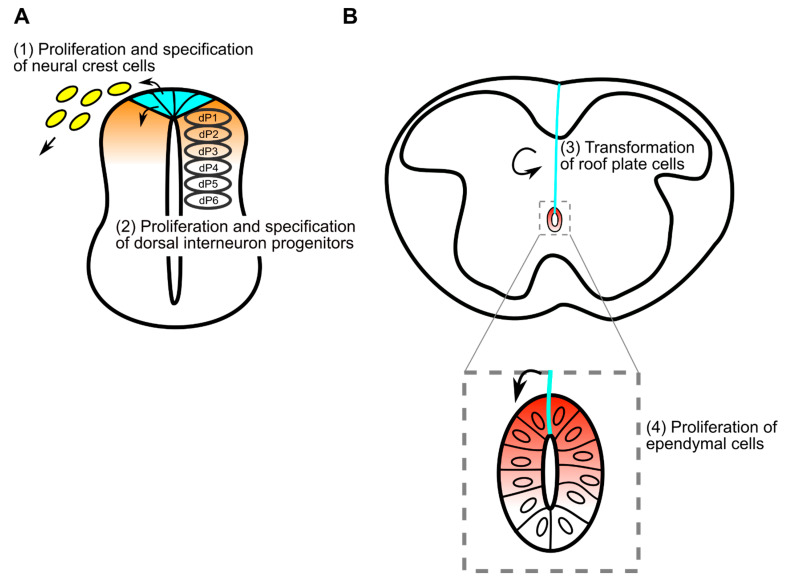
Multiple functions of Wnt ligands secreted from roof plate cells have multiple functions during spinal cord development. (**A**) Before stretching, roof plate cells exhibit a wedge-shaped form (cyan) and act as a source of neural crest cells (yellow), forming the dorsal organizing center. At this stage, Wnt signaling in roof plate regulates formation and fate determination of neural crest cells (1), as well as proliferation and specification of dorsal interneuron progenitors (2). In the dorsal spinal cord, Wnt signaling is likely to be activated in a gradual manner (orange). (**B**) After stretching, roof plate cells extend long processes (cyan) in contact with the central canal and pial surface. During this later stage, Wnt signaling in stretching roof plate cells regulates transformation of roof plate cells themselves (3) and proliferation of ependymal cells (4). In ependymal cells, Wnt signaling is likely to be activated in a gradual manner (red).

Since most *Wnt1-* and *Wnt3a-*double mutants die before E12.5 [19], genetic studies of *Wnt1-* and *Wnt3a*-deficient mutant mouse embryos yield no information regarding their roles in later stages. Thus, the function of Wnt signaling in the morphological transformation from roof plate cell to dmNes^+^RG was investigated by generating roof plate-specific conditional knock-out of the *Wls/Evi/Sprinter* gene, which is specifically required for secretion of Wnt proteins [80,81,82]. In normal embryos, prior to elongation of the apical processes of roof plate cells, the apical surfaces of roof plate cells are constricted, causing cell shape to become wedge-like at E10.5 (Figure 2A). At E13.5, when reduction of the lumen starts, apical processes of roof plate cells begin to elongate in parallel in a dorsoventral direction. Subsequently, these processes continue to elongate along the midline, accompanying reduction of the lumen, and nuclei of these cells become aligned on the midline [35,54] (Figure 2B,C). In *Wls* cKO embryos, the apical end of each process is also attached to the dorsal pole of the central canal, but these processes are not aligned along the midline. Rather, they extend laterally, slightly away from the midline. As a result, dorso-ventral nuclear alignment is impaired in mutant embryos. In addition, the bundle of processes of dmNes^+^RGs appears thinner and is frequently branched in mutant embryos, although the cytoskeletons are properly oriented [35]. These data suggest that mechanical tension in roof plate cells regulated by Wnt signaling may control the coordinated rearrangement of roof plate. However, since the number of roof plate cells is slightly increased in *Wls* cKO embryos at E13.5, alternative possibility that the rearrangement of roof plate cells is disrupted by crowding of roof plate cells cannot be excluded. Thus, Wnt secreted by roof plate cells is required for the change in morphology of these cells along the midline (Figure 3B).

## 5. Wnt Signaling in Development of Cells Surrounding the Central Canal

### 5.1. Wnt Signaling in Ependymal Cells

In the ependymal layer surrounding the central canal, Wnt/β-catenin signaling is activated [35,70]. This activation depends partly on Wnt secretion from dmNes^+^RGs, apical ends of which face the dorsal surface of the central canal. Actually, embryos in which *Wls* function is defective, specifically in stretched dmNes^+^RGs, exhibit a significant reduction in Wnt-active cells among ependymal cells at E15.5 and E18.5. Since this reduction is more severe in dorsal ependymal layer zone cells, it seems plausible that Wnt proteins from dmNes^+^RGs are specifically required for dorsal activation of Wnt/β-catenin signaling [35]. 

On the other hand, the Gene Expression Nervous System Atlas (GENSAT) Project revealed that several Wnt ligands, including *Wnt2b*, *Wnt3*, *Wnt7a* and *Wnt8b,* are expressed in spinal cord, except dmNes^+^RGs at E15.5 [83]. Thus, it also seems possible that Wnt proteins produced by some other cells, other than descendants of roof plate cells, are involved in activation of Wnt signaling in ependymal cells. In addition, since it was reported that a few Wnt-positive ependymal cells originated from the roof plate, it is also possible that these cells still maintain Wnt activity in the ependymal layer [70]. Actually, expression of *Wnt1* and *Wnt3a* is detectable in the ependymal layer shortly after birth, consistent with activation of Wnt/β-catenin signaling. In adults, many Wnt ligands, including *Wnt1*, *Wnt3a*, *Wnt5a* and *Wnt11*, are expressed in ependymal cells and expression of *Axin2*, a downstream target of Wnt/β-catenin signaling, is detected, indicating activation of Wnt/β-catenin signaling in these cells [70]. 

### 5.2. Proliferation of Ependymal Cells

In the spinal cord, a small number of ependymal cells constitutively proliferate [84], and these cells are concentrated dorsally [56]. Since Wnt/β-catenin regulates proliferation of neural progenitor/stem cells in the brain [85,86,87], it seems probable that this signaling is also involved in regulation of progenitor/stem cells in the spinal cord. A recent study showed that Wnt signaling actually appears to be involved in promoting proliferation of ependymal cells [35,70]. At E18.5, *Wls* cKO mutant embryos exhibited significant decreases in the frequency of Ki67-positive proliferating cells in the ependymal layer. Furthermore, this defect is more severe in the dorsal half, suggesting that Wnt secretion by dmNes^+^RGs is required for normal proliferation of ependymal cells in embryos [35] (Figure 3B).

### 5.3. Adult Spinal Cord

In adult mice, neurogenesis hardly occurs in the spinal cord. However, after injury to the spinal cord, ependymal cells proliferate and generate progeny that undergo multiple fates, suggesting that ependymal cells exhibit latent neural stem cell properties [55,61]. This injury-induced proliferation of ependymal cells is suppressed in *Wls* cKO mice, and as in embryos, cells in the dorsal half of the ependymal layer exhibit more severe defects [35]. Thus, Wnt secretion by roof plate cells is also required for proliferation of ependymal cells after injury to the spinal cord. 

Moreover, secretion of Wnt and expression of *β-catenin* in ependymal cells is required for proliferation of ependymal cells in postnatal and adult mice. Disruption of *β-catenin* or *Wls* in *Axin2*-expressing ependymal cells in adult mice significantly reduces the frequency of Ki67-positive proliferating cells in the ependymal layer [70]. Thus, activation of Wnt/β-catenin signaling and secretion of Wnt ligands is required for proliferation of ependymal cells in adult mouse spinal cord.

## 6. Conclusions and Future Perspectives

In conclusion, in early development of the spinal cord, the most dorsal cells act as a source of neural crest cells and constitute the dorsal organizing center, the roof plate, that regulates interneuron specification and proliferation. These functions are regulated by Wnt signaling in roof plate (Figure 3A). After neural specification, roof plate cells transform to dmNes^+^RGs, controlled by Wnt/β-catenin signaling. Moreover, Wnt ligands are still expressed in stretched roof plate cells and activate Wnt/β-catenin signaling in the ependymal layer (Figure 3B). Activation of Wnt/β-catenin signaling in ependymal cells promotes proliferation of neural progenitor and stem cells in embryonic and adult spinal cord. Thus, during spinal cord development, roof plate cells are a source of Wnt ligands and activate Wnt/β-catenin in themselves, as well as in surrounding cells that dramatically change morphology and function. 

Despite the function of roof plate cells in each stage of spinal cord development, regulatory mechanism of morphological change of roof plate cells by Wnt signaling still remain unclear. An interesting question for future studies is whether roof plate cells elongate their processes or are pulled by surrounding cells during transformation of roof plate cells. Given that the cytoskeleton regulates transformation of roof plate cells, a remaining challenge is to understand the mechanism by which Wnt signaling controls the cytoskeleton. In general, *Wnt1* and *Wnt3a* act as ligands to activate Wnt/β-catenin signaling, which directly regulates transcription but not cytoskeletal reorganization. Thus, understanding of the regulatory mechanism of roof plate transformation may provide a new insight into the Wnt signaling pathway in cell biology. 

In the adult spinal cord, Wnt/β-catenin signaling promotes cell proliferation in the ependymal layer in normal condition and after spinal cord injury. Pool of quiescent neural progenitor and stem cells in ependymal layer contribute to the regeneration in response to spinal cord injury. However, it remains unclear which type of the cells proliferate in ependymal layer in a Wnt/β-catenin signaling dependent manner and contribute to the regeneration after spinal cord injury. Elucidating the mechanism of cell proliferation and differentiation in the ependymal layer regulated by Wnt/β-catenin signaling may help to understand the mechanism of regeneration after spinal cord injury. 

## Data Availability

Not applicable.
